# HIV-Associated Tuberculosis

**DOI:** 10.1155/2011/585919

**Published:** 2010-09-13

**Authors:** Kogieleum Naidoo, Kasavan Naidoo, Nesri Padayatchi, Quarraisha Abdool Karim

**Affiliations:** ^1^CAPRISA Treatment Research Program, Centre for the AIDS Programme of Research in South Africa (CAPRISA), University of KwaZulu-Natal, 4013 Durban, South Africa; ^2^Department of Public Health and Family Medicine, University of KwaZulu-Natal, 4013 Durban, South Africa; ^3^Department of Epidemiology, Mailman School of Public Health at Columbia University, NY 10032, USA

## Abstract

The intersecting HIV and Tuberculosis epidemics in countries with a high disease burden of both infections pose many challenges and opportunities. For patients infected with HIV in high TB burden countries, the diagnosis of TB, ARV drug choices in treating HIV-TB coinfected patients, when to initiate ARV treatment in relation to TB treatment, managing immune reconstitution, minimising risk of getting infected with TB and/or managing recurrent TB, minimizing airborne transmission, and infection control are key issues. In addition, given the disproportionate burden of HIV in women in these settings, sexual reproductive health issues and particular high mortality rates associated with TB during pregnancy are important. The scaleup and resource allocation to access antiretroviral treatment in these high HIV and TB settings provide a unique opportunity to strengthen both services and impact positively in meeting Millennium Development Goal 6.

## 1. Introduction

Infection with *Mycobacterium tuberculosis* (MTB) remains one of the leading causes of morbidity and mortality globally [1]. The growing burden of multidrug-resistant (MDR) and extremely drug-resistant (XDR) cases of tuberculosis (TB) poses additional challenges to TB case management. Of the estimated 9.3 million new cases of TB that occurred in 2007, 1.37 million (15%) were co-infected with HIV. Sub-Saharan Africa accounted for 79% of the burden of TB-HIV co-infections, followed by South-East Asia (11%). In 2007, there were 456,000 TB-related deaths among HIV-positive patients accounting for 23% of the global HIV/AIDS mortality [2]. Globally, an estimated 33 million people are infected with HIV. HIV-positive people are about 20 times more likely than HIV-negative people to develop TB in countries with a generalized HIV epidemic [2]. Although South Africa is home to <1% of the global population, it accounts for 17% of the global burden of HIV infection and approximately one quarter of all HIV-TB co-infected patients [2, 3]. 

TB co-infection in HIV-infected pregnant women is emerging as a major non-obstetric cause of maternal deaths in sub-Saharan Africa [4]. According to data from two studies at a major tertiary hospital in KwaZulu-Natal (the epicenter of the pandemic), Khan et al. [5] in 2001 demonstrated a 32-fold increased risk of death among TB-HIV co-infected pregnant women compared to those without HIV-infection, and Ramogale et al. [6] in 2007 reported that the majority of deaths among HIV-infected pregnant women were associated with TB co-infection. TB is also a major cause of morbidity in HIV-infected children, with HIV-infected children having a 20–25-fold higher incidence of TB than HIV-uninfected children, with an overall TB incidence in South African HIV-infected children of 9.2% (95% CI: 0.14–0.97) [7–9].

The intersection of the TB and HIV epidemics and the challenges and opportunities they provide for addressing both epidemics is the focus of this paper. 

## 2. Diagnosing TB in HIV-Infected Patients

TB is the most common presenting opportunistic infection among HIV-infected patients, who remain at high risk for TB throughout the course of their disease. HIV-infected patients on HAART have a TB incidence rate of 4.6 cases/100 patient-years, approximately 10-fold higher than TB incidence rates in HIV-negative patients in the same community [10]. Early identification of TB suspects, with prompt triage into TB diagnostic and treatment services, are beneficial in improving overall clinical outcomes in co-infected patients, as well as in reducing TB transmission from undiagnosed and untreated patients. Additionally, HIV-infected patients who screen negative for TB can be assessed for eligibility for isoniazid (INH) preventive therapy. Notwithstanding these benefits, HIV-TB co-infected patients have a higher frequency of smear negative TB sputum tests [11] and are four times more likely to have extra-pulmonary TB and atypical as well as nonspecific radiologic features of TB. Thus, clinical diagnosis of pulmonary tuberculosis (PTB) in patients co-infected with HIV remains a challenge especially in settings where there are limited clinicians and radiological facilities to confirm a TB diagnosis [12–14]. Further, HIV-TB co-infected patients have varied clinical presentation of TB, ranging from the classic signs of TB infection of cough, weight loss, fever, and drenching night sweats to nonspecific signs and symptoms [15, 16]. Diagnosis of drug-resistant TB remains a challenge in many high-burden TB settings, largely as a result of a combination of poor laboratory capacity and the absence of widespread programmatic drug susceptibility testing. Mortality rates in excess of 80% have been reported for patients with HIV and XDR-TB, in the context of an XDR-TB outbreak, and have been attributed to diagnostic delays and lags in instituting appropriate therapy [17]. Recent years have seen an investment of research effort into developing new assays with increased sensitivity and specificity, shorter turn-around times, with greater suitability for high burden resource-constrained settings, and potential value in HIV-infected individuals (Table 1). A study by Dheda et al. [18], showed urine-LAM positivity to be significantly associated with HIV positivity (*P*  =  .007), with sensitivity significantly higher in HIV-infected compared to uninfected patients (*P*  =  .001), and in HIV-infected participants with a CD4 < 200 cells/mm^3^, compared to those with CD4 counts above 200 (*P*  =  .003). In this particular study, urine-LAM remained highly specific for TB both in HIV-uninfected and in HIV-infected patients irrespective of CD4 counts. Twenty-five percent of smear-negative but culture-positive HIV-infected patients with a CD4 < 200 cells/mm^3^ were positive for urine-LAM [18]. It is important to note that T-cell-based assays such as the interferon gamma release assays, which include the T-Spot-TB and QFT Gold-in-tube, are not useful in the diagnosis of active TB because these tests do not distinguish between latent and active TB.

## 3. Enhancing TB Diagnosis in HIV-Infected Patients and Knowledge of HIV Status in TB Patients and Prophylactic Use of Isoniazid

To overcome diagnostic infrastructure and human resource constraints, a TB symptom screening questionnaire is increasingly being utilised to more effectively and efficiently screen for TB [26]. Data from nine ICAP ARV treatment initiation sites in the Eastern Cape, where infrastructure and human resource capacity are extremely limited have provided compelling evidence for the utility of this screening tool. ARV treatment sites can utilise this screening tool to maximise opportunities for TB screening in HIV-infected patients and TB services can include provider-initiated counselling and testing (PIT) services to increase knowledge of HIV status in TB patients, linked to CD4 testing and/or clinical staging of HIV disease in patients testing HIV positive, and potentially provide earlier access into AIDS care and treatment services [27]. The World Health Organization (WHO) currently recommends PIT as a standard of care for all patients with signs and symptoms of TB [28]. South Africa has, since the 1st April 2010, taken this a step further and now emphasises HIV counselling and Testing (HCT) as part of opportunistic health promotion [29]. Given that South Africa has an HIV TB co-infection rate that exceeds 69%, this is a significant policy shift for enhancing HIV survival and impacting the TB epidemic in this setting [30].

## 4. Integration of HIV, TB, and MDR-TB Services

Though intertwined, HIV and TB are treated in separate programs and facilities. TB clinics have been slow to implement HIV care and the introduction of antiretroviral therapy (ART), which has resulted in the establishment of separate HIV clinics, that often do not focus on TB co-infection [31, 32]. In countries with dual HIV-TB epidemics, the provision of HIV testing to TB patients and the provision of CD4+ count assays to those who test HIV-positive are a cost-efficient approach in identifying co-infected patients in need of ART [31]. One strategy to improve outcomes for HIV-TB patients is to utilize existing TB-care facilities and combine HIV care with TB care. TB clinics already have an established infrastructure, drug, and patient management systems. Treatment of both diseases requires addressing adherence to therapy and monitoring for side effects, toxicities, and treatment failure. Up until early 2010, uncertainty existed as to the best time to initiate HAART in HIV TB co-infected patients. Issues of high mortality rates in co-infected patients not initiated on ART were offset by concerns of concomitant treatment of both diseases including increased frequency of immune reconstitution inflammatory syndrome (IRIS); potential interaction between rifampicin and NNRTI and PI drugs; pill burden, adherence, and overlapping toxicity. Data from the Starting Antiretroviral Therapy at Three Points in Tuberculosis (SAPiT) trial provided empiric evidence for integration of HIV and TB care, with a reduction in all-cause mortality of 56% among co-infected patients with CD4+ counts< 500 that integrated HIV and TB treatment compared to those that deferred ART initiation until after TB treatment was complete. Additionally, patients that had integrated HIV and TB treatment had both favourable TB outcomes and reduced incidence of IRIS. The question of whether the most optimal time to initiate ART is during the intensive or continuation phase of TB treatment remains unanswered [33]. Thus far, studies have demonstrated poorer MDR-TB and HIV treatment outcomes in dually infected patients. MDR-TB HIV co-infection has been described as the “perfect storm”, as it is associated with extremely high mortality and lack of adequate second line anti-TB therapy. Complexities in managing MDR-TB in HIV include the availability of rapid drug susceptibility testing, prevention of nosocomial transmission to patients and HCW, potentiated drug toxicity, ART drug interactions with MDR-TB treatment, and a high pill burden [34, 35]. Publications from South Africa by O'Donnell et al. [36] and Dheda et al. [17] have demonstrated improved outcomes with the integration of antiretroviral therapy with MDR and XDR therapies, with respect to overall reduction in mortality rates and better outcomes in co-infected patients that integrated therapies for HIV and drug-resistant TB.

## 5. Immune Reconstitution Inflammatory Syndrome (IRIS)

TB IRIS results from either deterioration of TB while on anti-TB treatment, known as paradoxical TB IRIS, or a new presentation of previously subclinical TB referred to as unmasking TB IRIS. Mycobacteria account for approximately 40% of all cases of infective IRIS in patients initiated on ART [37–41]. The frequency of paradoxical TB-IRIS range from 8% to 43% [42–45] and is dependent on background TB prevalence rates. In contrast, literature describing the prevalence and presentation of unmasking TB IRIS is more limited [46–48]. The common consensus from clinical studies suggests that low CD4+ cell counts are associated with an increased incidence of IRIS [49, 50]. The introduction of ART early in TB treatment is an additional risk factor for IRIS. A South African study indicated a 12% overall incidence of TB IRIS in HIV-TB co-infected patients, with an IRIS incidence of 32% when ART was initiated within 2 months of TB diagnosis and an IRIS incidence of 70% if ART was initiated within a month of starting TB therapy [51]. A meta-analysis of IRIS studies by Muller et al. [50] suggests that both frequency of IRIS events and the high early mortality in ART programs in resource-limited settings could be prevented by starting ART earlier prior to the risk of opportunistic infections. 

## 6. Prophylaxis in HIV-TB Co-Infected Patients

Data from the Development of AntiRetroviral Therapy in Africa (DART) trial have demonstrated that cotrimoxazole prophylaxis reduced mortality in patients with HIV infection on ART for up to 72 weeks regardless of CD4 count status, with mortality similarly reduced in patients with a current CD4 cell counts above and below 200 cells/*μ*L [52]. Notwithstanding the absence of definitive evidence from randomised controlled trials on optimal duration of IPT in HIV-infected patients, data from several observational studies demonstrate that INH Preventive Therapy (IPT) is both cost effective and beneficial [53–55] through combating low bacillary load latent TB which serves as a reservoir for possible recurrent disease [56]. 

Concurrent administration of ART and IPT has demonstrated a TB risk reduction of 76–89% in observational studies from both South Africa and Brazil [54, 55]. Given the challenges in diagnosing TB in HIV-infected patients, the potential of unintentionally treating active TB with monotherapy and thereby contributing to drug resistance is very real especially in settings with inadequate sputum, radiology, and tuberculin skin testing resources for the exclusion of active TB [57]. Significantly, despite the need to exclude active TB prior to the administration of IPT, many people die of TB without having received either IPT or any appropriate treatment for their unrecognised TB [58]. 

The protection offered by IPT to those infected with HIV may depend on a number of factors including degree of immune suppression of the individual, duration of IPT, adherence to and potency of the regimen, as well as the general risk of reinfection in that setting [56, 59]. Data from randomised controlled trials using 6–12 month IPT in HIV positive patients demonstrate reductions in risk of active TB by about a third compared to those on placebo, with reductions of up to two-thirds in those who tested tuberculin skin test (TST) positive [56, 57, 60]. Additionally, a Botswana trial that initiated 1,006 patients on continuous IPT demonstrated that IPT was 92% effective in reducing TB in TST-positive patients [61]. An analysis of published studies on the treatment of latent TB infection by Akolo et al. [62] demonstrated that TST negative individuals seem not to benefit from IPT. While more studies are required to establish optimal duration of IPT in HIV-infected patients in high-burden TB settings, current data suggest optimal benefit from IPT when used for a period of 9 months post TB therapy [63]. Current recommended dosages for IPT include 300 mg daily for 6–9 months in adolescents and adults or alternatively 800–900 mg twice weekly if supervised treatment is feasible [64–67]. 

While toxicity from coadministration of IPT and HAART is valid, the likelihood of stopping therapy as a result of adverse events has to date been low. The risk of adverse events was higher with rifampicin and pyrazinamide-containing regimens than INH monotherapy [56]. The feasibility of implementing IPT into HIV care programmes has been demonstrated in many African settings [27]. INH adherence education and motivation, with monitoring of ongoing adherence and toxicity can be readily integrated into HIV care programmes. There are several benefits of instituting IPT to patients in HIV wellness and pre-ART readiness programmes, including protection from TB-associated morbidity and mortality, and the incentive for regular clinic attendance for patients.

## 7. Recurrent TB

Recurrent TB accounts for the majority of TB cases in countries with a high TB incidence rate [2] with HIV infection a major risk factor for TB recurrence [68–70]. The WHO Guidelines defines recurrent TB as the diagnosis of active TB in patients previously treated for TB and were subsequently declared cured or whose treatment was completed. Data from South Africa indicate an association between high TB incidence and ART initiation at CD4 < 200 cells/mm^3^ or at WHO Stage 4 HIV disease [71]. HIV-infected patients on HAART have a TB incidence rate of 4.6 cases/100 patient-years, approximately 10-fold higher than the TB incidence rates of HIV-negative patients in the same community. A study of South African gold miners demonstrated that TB recurrence was 5 times more likely in HIV-positive patients [72]. The “risk of TB relapse” remains a significant factor in determining optimal duration of anti-TB therapy. Current guidelines suggest a 6-month rifamycin-based regimen as suitable irrespective of HIV status, yet to date, two randomized clinical trials suggest otherwise [73, 74]. One RCT compared 12-month standard rifampin-based regimen to a 6-month regimen and found lower recurrence rates at 18 months in the group with an extended TB therapy duration [74]. The second RCT which compared standard short course therapy in both HIV-infected and uninfected groups showed that rates of recurrence in HIV-infected individuals were only reduced when combined with 1 year of continued INH therapy post short-course TB therapy completion [73]. Of note, is that both trials predated the ART era. There is a paucity of data on incidence rates and risk factors for recurrent TB in an HIV-infected population on ART. TB scarring, cavities, regimens used for the initial episode, and a low CD4 count are some risk factors associated with recurrent TB. There has been considerable interest in the timing of HAART and its capacity for immune restoration. Irrespective of the level of TB burden in different settings, HAART has consistently been shown to substantially reduce the risk of TB within HIV cohorts by 70–90%. It is, however, hypothesised that partial immune restoration resulting from ART creates a persistently heightened risk for recurrent TB [75, 76], which is in contrast to the role of HAART in community TB control, as patients on HAART survive longer, yet they remain at a chronic heightened risk for TB infection [77]. With increasing numbers of patients on ART spending long hours in inadequately ventilated waiting rooms in AIDS clinics and where limited infection control activities are implemented, the impact of recurrent TB especially with drug-resistant TB needs to be urgently investigated. Scaleup of ART services remains an essential strategy in TB control. Declining incidence density rates of TB in the first five years of HAART have been demonstrated by Lawn et al. However, patients with CD4 < 100 cells/ml and more advanced pretreatment immune suppression remain at heightened risk for recurrent TB [77].

## 8. Enhancing Infection Control for TB in ARV Clinics

Given the high rates of undiagnosed TB in AIDS patients and the excess mortality associated with HIV-TB co-infection, HIV clinics should pay particular attention to infection control including good ventilation to minimise further TB risk acquisition in AIDS patients. The Tugela Ferry XDR-TB outbreak in rural South Africa brought to public attention the consequences of nosocomial TB transmission in high HIV burden settings and has also highlighted the risk of acquiring multi-drug-resistant TB in health care workers and in the general public. A 3-tier approach for TB-infection control has been developed by the WHO for application in all settings and includes administrative and environmental controls and personal protection. The proposed interventions are simple and feasible and can be customised further based on available resources [78]. Administrative controls include (i) early identification and separation of infectious patients, (ii) controlling infectiousness by educating patients on proper cough etiquette and respiratory hygiene, and (iii) limiting time patients spend in health care facilities through decreasing hospital stays and increasing outpatient TB treatment [79]. Environmental controls include (i) encouraging natural and mechanical ventilation and (ii) upper-room ultraviolet light. Natural ventilation could be as simple as opening a window and a door to create cross ventilation. However, mechanical ventilation can be complicated and expensive as the installation of split vent cooling and air exchange systems, or as simple as installing ceiling and upright fans. UV light, if installed properly, has been shown to damage the MTB organism without causing harm to patients [80–82]. Low-cost sputum booths are increasingly being erected within clinics as an environmental infection control measure in high burden TB settings [83]. Personal measures include (i) staff use of particulate respirators such as the N95 respirator though effectiveness is dependent on staff compliance, additional training, as well as fit testing in ensuring proper use and (ii) provision of surgical masks for patients to create a mechanical barrier to the spread of infectious droplet nuclei [84–86].

## 9. Conclusion

Much has been learnt about HIV-related TB and much remains to be learnt. Research efforts need to focus on protective TB vaccines for immuno-compromised populations, new first- and second-line TB drugs, point of care diagnostics for TB and TB IRIS, including strategies to optimise clinical outcomes in patients co-infected with HIV, and drug-resistant TB. The intersection of the HIV and TB epidemics provides a unique opportunity to strengthen TB and HIV services with substantial morbidity and mortality gains at the individual and population level. This level of public health benefit in the current fiscal climate and growing disease burden is critically important. The integration of HIV and TB services could serve as an important role model for other service delivery. Significantly, reducing and better managing TB in HIV-infected persons could make a substantial contribution to countries reaching their MDG health goals.

## Figures and Tables

**Table 1:** Newer Diagnostic Technologies for TB.

Technology	Turnaround time	Sensitivity gain	References
Ziehl Neelsen	2-3 days		[19]
Solid Culture	30–60 days	Baseline	

Liquid Culture	15–30 days	+10%	
Rapid Speciation		Compared to LJ	

Line Probe Assay 1st line Rif and INH (e.g., INNOLiPA Rif.TB and the GenoTyp MTBDR)	2–4 days	Currently smear positive only	[20, 21]

Integrated Nucleic Acid Amplification Tests (INH and Rif only) GeneXpert MTB/RIF	90 minutes	+40% compared to ZN	[22]

The Microscopic-Observation Drug-Susceptibility (MODS)	7 Days	+14% compared to LJ	[23]

T-cell-based Assays (Interferon-*γ*-based assays)	16–24 hrs (if not batch run)	Overall sensitivity gain unknown Sensitivity: 76% for QTF-G, and 90% for Elispot, unable to distinguish latent from and active TB, role in HIV infection uncertain	[24]

LED-based Flourescent Microscopy	1-2 days	+10% Compared to ZN	[19]

TB Antigen Detection (Lipoarabinomannan –LAM Assay)	3-4 hours Point of care testing available soon	Overall sensitivity gain unknown 52% sensitivity in HIV+ patients, specificity 89%	[18, 25]
